# Exploring Hidden Connections: Endophytic System and Flower Meristem Development of *Pilostyles berteroi* (Apodanthaceae) and Interaction with Its Host *Adesmia trijuga* (Fabaceae)

**DOI:** 10.3390/plants13213010

**Published:** 2024-10-28

**Authors:** Ana Maria Gonzalez, María Florencia Romero, Héctor A. Sato

**Affiliations:** 1 Instituto de Botánica del Nordeste (UNNE-CONICET), Facultad de Ciencias Agrarias, Universidad Nacional del Nordeste, Corrientes PC 3400, Argentina; mariafloromero@gmail.com; 2 Centro de Estudios e Investigaciones Botánicas, Facultad de Ciencias Agrarias, Universidad Nacional de Jujuy, Jujuy PC 4600, Argentina; hector.a.sato@fca.unju.edu.ar

**Keywords:** CLSM, chimera, endoparasites, holoparasitic, SEM, sinkers, tracheoids, wood

## Abstract

*Pilostyles*, an endoparasitic genus within the Apodanthaceae family, grows inside host stems with flowers and fruits being the only external manifestations. Previous studies of *P. berteroi* growing on *Adesmia trijuga* provided limited details of the endophyte and omitted the origin of flowers and sinker structure. This study, using classical methods of optical microscopy applied to the analysis with scanning electron microscopy and confocal laser scanning microscopy, expands the understanding of the *P. berteroi*/*A. trijuga* complex. We find that *P. berteroi* develops isophasically with its host, forming endophytic patches between the host’s secondary phloem cells. The parasitized *Adesmia* stem’s cambium primarily produces xylem parenchyma, with limited vessel production and halting fiber formation. The radial polarization of endophytic patches led to the formation of floral meristems. Flowers develop endogenously and emerge by the breakthrough of the host stem. Flowers are connected to the host cambium via chimeric sinkers, combining *P. berteroi* parenchyma and tracheoids with *Adesmia* vessels. Unlike previous studies that show uniformity among *Pilostyles* species, our analysis reveals new insights into the structural interaction between *P. berteroi* and *A. trijuga*.

## 1. Introduction

Researchers have always been fascinated by parasitic plants, their life cycles, complex and bizarre structures, physiology, and interaction with their hosts [1,2]. Due to the strange and cryptic nature of this life form, they have been the subject of multiple studies, especially in recent years [3,4,5,6,7,8,9,10,11,12].

Traditional classifications divide parasitic plants into hemiparasites, capable of photosynthesis, and holoparasites, achlorophyllous plants that are totally dependent on their host [1]. Other classifications present functional groups, considering—in addition to the traditional characters—others such as type of haustorium, place of connection with the host, growth form, and type of germination, among others [5,6,8,13]. Thus, Tesitel [13] proposes four functional groups: root hemiparasitism, root holoparasitism, stem parasitism, and endophytic parasitism. Recently, five functional groups of parasitic plants focused on life history were identified: euphytoids, mistletoes, parasitic vines, obligate root parasites, and endoparasites [5].

Among the variants of parasitism among plants, endoparasites represent the most advanced and specialized form of holoparasitism [1,2,5,13,14]. Endoparasites are emphasized as a distinct functional group due to the “predominance of an endophytic phase in their life cycle” [13]. These have a unique vegetative structure, as they are completely enclosed in the stems of their host, where they grow like fungi [6,13,15,16]. The only visible signs of the parasite are the flowers and fruits that emerge from the host stem through the bark.

Endoparasites are present in families such as Apodanthaceae, Cytinaceae, Mitrastemonaceae, and Rafflesiaceae [5,16,17]. Alternatively, they may appear as shoots in various endophytic mistletoe genera within the Santalales [5,13].

The family Apodanthaceae Tiegh. ex Takht., order Cucurbitales, consists of endoparasites, including two genera and thirteen species [17,18]. The genus *Apodanthes* Poit. is monospecific and can only be found in the tropical rainforests of the New World. On the other hand, the genus *Pilostyles* Guill. consists of twelve species and has a broad distribution that spans arid regions in North, Central, and South America, Iran, Iraq, Syria, subtropical East Africa, and southwestern Australia [17,19,20,21,22]. *Pilostyles* species grow inside the stems of Fabaceans and Salicaceans [17,23]. *Pilostyles berteroi* Guill. was collected from *Adesmia trijuga* Gillies ex Hook. and Arn. during field trips in NW Argentina.

The endophytic body of *Pilostyles* is simple, without typical organs, such as roots, stems and leaves, presenting only a mycelial-like system formed of parenchyma cells and tracheary elements that invade the host stem [14,23,24,25,26,27,28,29,30,31,32]. The development of morphology, anatomy, and flowers has been documented in various Apodanthacean species [18,25,28,30,33,34,35,36,37,38]. The only study of *P. berteroi* growing on *A. trijuga*, focused on the general structure of the staminate and pistillate flowers, proposed a mechanism of emergence through the cortex of the Fabacean host [28]. Based on previous descriptions of *P. blanchetii* [Gardner) R. Br. and *P. haussknechtii* Boiss. [25,26,27], Kummerow [28] provides an overview of the endophytic body, describing the parasitic tissue as large, plasma-rich cells with a prominent nucleus and two nucleoli, distinguishing them from host cells. In the genus *Pilostyles*, the connections between the flowers and the host’s xylem are called sinkers [29]. Kummerow [28] notes that he was unable to describe the sinkers with the material he had available, and the study also does not explore the origin of the flowers of *P. berteroi*. A comprehensive morphological and anatomical analysis of the sterile and fertile cycles in staminate and pistillate flowers of *P. berteroi*, including gamete formation, fertilization, and seed development, has been undertaken [38].

The host of *P. berteroi* is *Adesmia trijuga*, a spiny fabacean plant native to Argentina and Chile that grows in semi-arid or arid mountainous regions at elevations of 1100–3000 m [39,40,41]. Anatomical studies of *A. trijuga* have focused on the leaves [42] and some secretory structures [43]. The wood anatomy has only been described in *A. boronioides* Hook [44], *A. rahmeri* Phil., and *A. spinosissima* Vogel [45]. Within the Apodanthaceae family, the impact of holoparasite growth inside the host has been studied in *P. blanchetii* [Gardner) R. Br. in various species of *Mimosa* [23].

Given the scarce knowledge of this particular species of endoparasitic plant, the objectives of this study are as follows: (1) to provide a comprehensive description of the anatomical differences between healthy *A. trijuga* stems and those parasitized by *P. berteroi*; (2) to re-evaluate the characteristics of the endophyte; (3) to elucidate the process of flower primordia formation; and (4) to describe the structure and cellular composition of the sinkers, comparing these characteristics with those of other *Pilostyles* species analyzed so far.

## 2. Results

### 2.1. Pilostyles berteroi–Adesmia trijuga System

**Figure 1:**
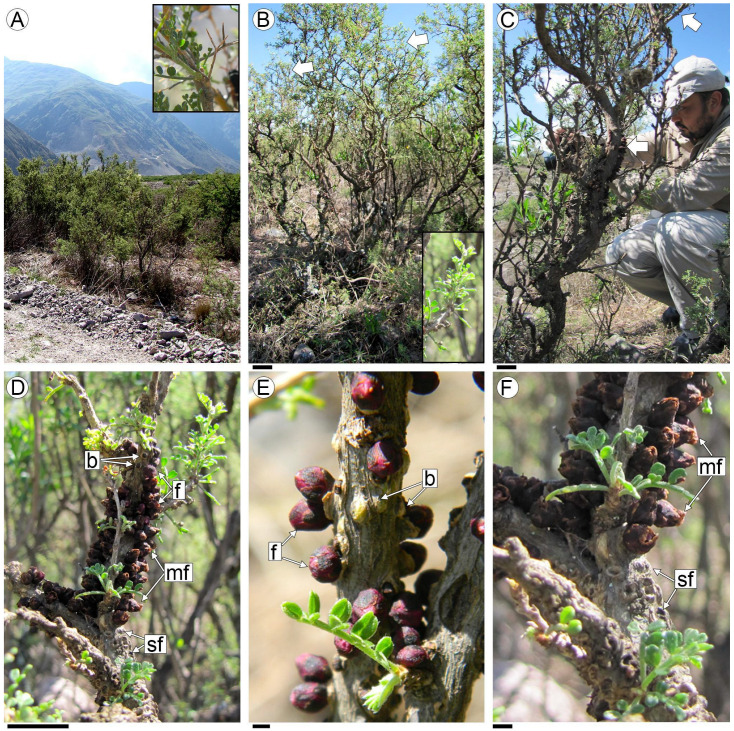
Specimens of *A. trijuga*. (**A**) General aspect of the landscape with *A. trijuga* plants; (**B**) *A. trijuga* plants, where the zone of stem secondary structure is indicated by arrows and detailed in the inset; (**C**) *A. trijuga* plant parasitized by *P. berteroi* (arrows indicate the parasite’s flowers); (**D**–**F**) *P. berteroi* flowers emerging from host stems; (**E**) Detail of buds and flowers at anthesis; (**F**) Basal region with older staminate flowers and scarring from previous year’s flowers. Abbreviations: b: flower buds, f: flowers in blossom, mf: mature flowers; sf: scars of flower or fruits. Scales: (**B**,**C**): 10 cm; (**D**): 1.5 cm; (**E**,**F**): 0.5 cm.

*Adesmia trijuga* is a spiny, microphyllous shrub. Its stems are characterized by the rapid development of secondary growth, which appears only a couple of centimeters from the apex of the branches, and is identified by the presence of peridermis (Figure 1A,B). External signs of parasitism by *P. berteroi* on the stems of *A. trijuga* are the flowers and the scars left by abscised staminate flowers or fruits from previous seasons (Figure 1C,F).

The flowers break through the bark on stems with secondary growth, approximately 30–40 cm from the apex of the branch (Figure 1D). These unisexual flowers are distributed across different branches of the same *A. trijuga* specimen. They emerge gregariously through the bark in longitudinal rows (Figure 1D,F). The flowers of *P. berteroi* appear acropetally on the branches of *A. trijuga*. In the upper half of the infested area, flower buds are intermixed with blooming flowers (Figure 1D,E). At the base of the infested area, the senescent flowers or fruits and the scars from the flowers of the previous season are visible (Figure 1F). Thus, each year, the appearance of flowers progresses towards the apex of the branches of *A. trijuga*.

### 2.2. Anatomy of Non-Parasitized Stems of Adesmia trijuga

**Figure 2:**
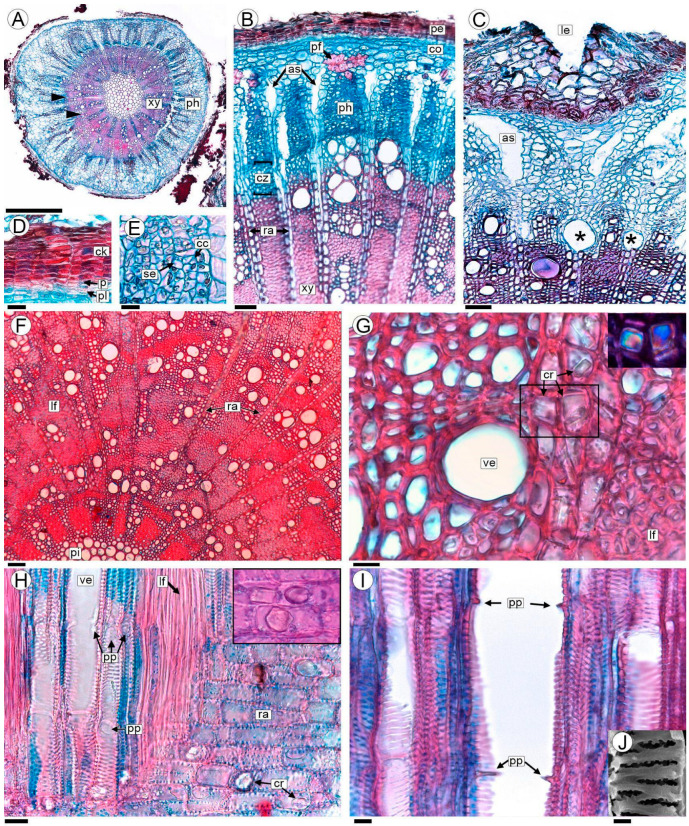
Anatomy of the non-parasitized stem of *A. trijuga* in transversal (**A**–**G**), and radial longitudinal sections (**H**,**I**) are analyzed with a light microscope (LM). (**A**) Transection of 2-year-old non-parasitized stem; (**B**,**C**) phloem and cambial zone in a stem with vessels in the process of formation (*); (**D**) periderm; (**E**) secondary phloem; (**F**) wood anatomy showing its diffuse porosity; (**G**) detail of vessels, paratracheal parenchyma and libriform fibers; the crystals in the area indicated with a square are shown in the inset (polarized light); (**H**) vessels, libriform fibers and rays; crystals in the inset; (**I**) vessels showing simple perforation plates; (**J**) detail of vestured pits. Abbreviations: as: air spaces; cc: companion cells; ck: cork; co: cortex; cr: prismatic crystals; cz: cambial zone; lf: libriform fibers; le: lenticels; p: phellogen; pe: periderm; pf: primary phloem fibers; ph: secondary phloem; pi: pith; pl: phelloderm; pp: simple perforation plate; ra: rays; se: sieve elements; ve: vessels; xy: xylem. Scales: (**A**) 0.5 mm; (**B**,**C**,**F**) 50 µm; (**D**,**H**) 20 µm; (**E**,**G**,**I**) 10 µm; (**J**) 2 µm.

The stems with secondary growth are characterized by the presence of a continuous periderm, composed of either phellogen or the cork cambium; phellem or “cork” to the outside and phelloderm to the inside (Figure 2A,B). The phellogen and phelloderm are unistratified and the cork varies from three to more than ten cell layers (Figure 2B,D). All periderm cells form very ordered stacks in radial alignment with no intercellular spaces (Figure 2B,D). Lenticels are abundant and large relative to the stem diameter (Figure 2C).

The cortex consists of parenchymatous cells with very thin walls. The primary phloem has small caps of thickened-walled fibers (Figure 2B). The secondary phloem is characterized by sieve elements and their companion cells (Figure 2B–E). The phloem rays are 2–3 cells thick at the cambium level and within older phloem portions they expand and separate to form dilated rays with air spaces. The phloem is thus formed into long triangular extensions (Figure 2B,C).

The secondary xylem exhibits indistinct growth rings characterized by vague, imprecise, or absent boundaries (Figure 2B,F). In certain areas, these boundaries may be marked by marginal bands of parenchyma or thick-walled libriform fibers that help delineate the limits of the growth rings (Figure 2F). The wood is diffuse-porous, the vessels are solitary or in tangential bands, and commonly in clusters. The vessels are circular in outline and small-sized, with a 24 (13.8–34.2, SD ± 10.2) µm diameter (Figure 2F,G). The perforation plates are simple; the intervessel vestured pits scalariform to opposite; the vessel-ray pits are similar in shape and size to the intervessel pits (Figure 2H–J). Libriform fibers have simple to minutely bordered pits, and are very thick-walled (Figure 2G,H). Parenchyma is predominantly paratracheal and is also in 2–5 cell-wide marginal bands. Rays are 1–3 cells wide, and prismatic crystals are present (Figure 2B,F–H). The pith is composed of thick-walled parenchyma cells. The elements of the primary xylem are indistinct (Figure 2F).

### 2.3. Parasitized Stems

**Figure 3:**
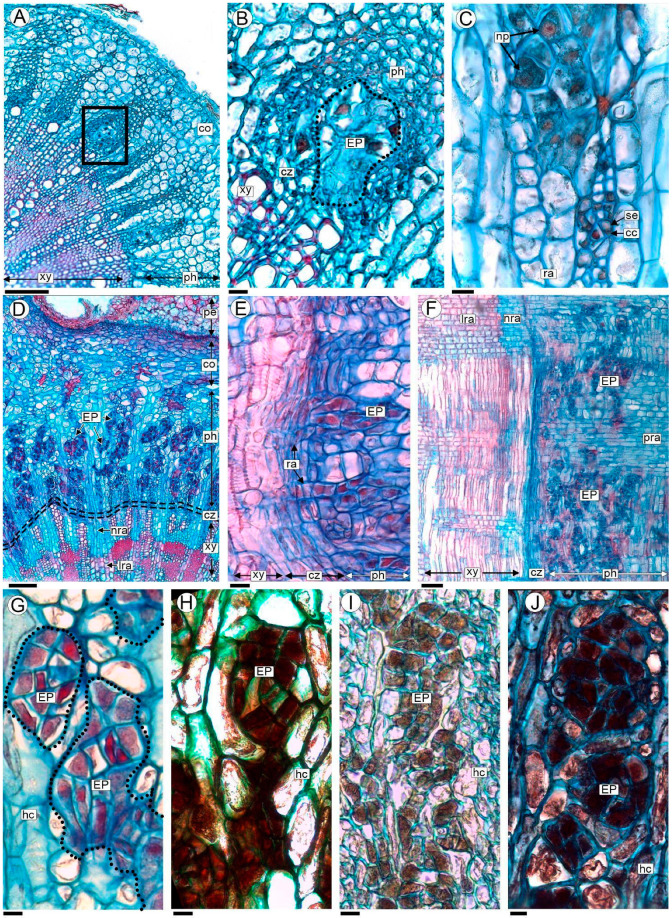
Stems of *A. trijuga* parasitized by *P. berteroi* in transverse (**A**–**D**,**G**–**J**), and radial longitudinal section (**E**,**F**), analyzed with LM. (**A**) Stem of *A. trijuga* with early stages of parasite development; the first endophytic patches (EPs) of *P. berteroi* cells are seen in the phloem (box); (**B**) detail of an EP of *P. berteroi* corresponding to the box in (**A**); (**C**) detail of EPs of *P. berteroi* and cells of secondary phloem of *A. trijuga*; (**D**–**F**) parasitized stems showing the presence of numerous EPs that occupied most of the secondary phloem. (**G**) EP stained with Safranin–Astra blue; (**H**–**J**) EP subjected to histochemical tests, combined with Astra blue to enhance cell wall identification: (**H**) lugol (black) for starch, (**I**) ferric chloride (brown) for phenol groups, (**J**) sudan black (blue–black) for lipids. Abbreviations: cc: companion cells; co: cortex; cz: cambial zone; hc: host cells; EP: endophytic patches; lra: lignified rays; nra: non-lignified rays; np: parasite nuclei; pe: periderm; ph: secondary phloem; pra: phloem rays; ra: rays; se: sieve elements; xy: secondary xylem. Scales:  (**A**,**F**) 100 µm; (**B**,**C**,**E**,**G**–**J**) 10 µm; (**D**) 50 µm.

The first evidence of *P. berteroi* cells can be found in the phloem of stems undergoing secondary growth (Figure 3A and Figure S1A). The endophytes of *P. berteroi* grow vegetatively, forming compact clusters that develop into patches or distinct areas within the elements of the secondary phloem of *A. trijuga* (Figure 3B,C). We designate this arrangement as endophytic patches (EPs). The primary phloem and cortex regions are not invaded by holoparasite cells (Figure 3B,C and Figure S1B).

In older stems of *A. trijuga*, multiple EPs were observed in the secondary phloem, forming a mycelial-like structure representing *P. berteroi*’s vegetative body (Figure 3D–F and Figure S1A–F). The EP’s cells extend into the cambial region, where they are preferentially observed at the level of the initial radial cells (Figure S1F). The differentiation of the secondary xylem is modified; the vascular cambium produces mainly parenchyma cells inwards, production of tracheary elements is restricted to certain areas and libriform fiber formation is arrested (Figure 3D–F and Figure S1B–F). However, the EPs were never observed penetrating vessels (Figure 3D–F). The secondary phloem cells that are formed by the cambium during the *P. berteroi* infestation do not show any signs of variation in structure (Figure 3D–F and Figure S1A,B).

All *P. berteroi* cells in EP are parenchymatic and isodiametric, with uniformly thin walls and no intercellular spaces (Figure 3B,C). Endophytic cells have a prominent, more or less centrally located nucleus (6.15–8.25 µm), often with two nucleoli. The EP´s cells have a dense cytoplasm which distinguishes them from the surrounding host tissue (Figure 3C,G). The results of the performed histochemical stains indicate the presence of starch (detected with Lugol), lipids (Sudan black) and tannins (Ferric chloride, Figure 3H–J).

### 2.4. Origin of Pilostyles’ Flowers

**Figure 4:**
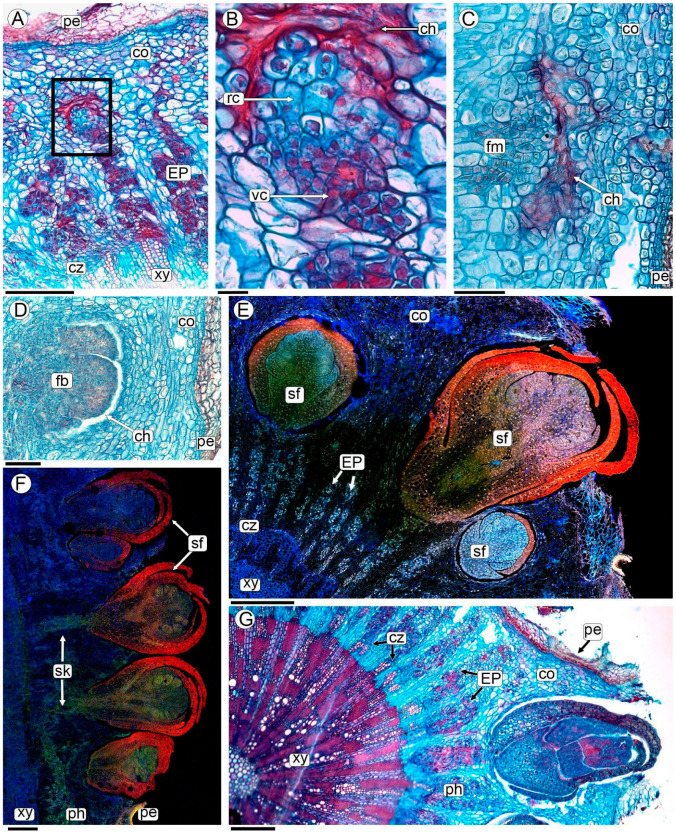
Flower development of *P. berteroi* in stems of *A. trijuga*, analyzed with LM (**A**–**D**,**G**), and CLSM for autofluorescence (**E**,**F**). (**A**,**B**,**E**,**G**) Cross-sections and (**C**,**D**,**F**) radial sections of infested *A. trijuga* stems. (**A**) Origin of the *P. berteroi* floral meristem from an EP (box); (**B**) Close-up of box in (**A**) showing the polarity of EP with vegetative and reproductive cells surrounded by collapsed host cells; (**C**) floral meristem; (**D**) meristem with first bract whorl; (**E**–**G**) *P. berteroi* staminate flowers at various stages of development, with some already emerging through the periderm of the stem of *A. trijuga*. Abbreviations: ch: collapsed host cells; co: cortex; cz: cambial zone; EP: endophytic patches; fb: floral bud; fm: floral meristem; pe: periderm; ph: phloem; rc: reproductive cells; sf: staminate flower; sk: sinker; vc: vegetative cells; xy: xylem. Scales: (**A**) 200 µm; (**B**) 20 µm; (**C**) 100 µm; (**D**,**E**) 200 µm; (**F**,**G**) 1 mm.

*Pilostyles berteroi* flower initiation occurs entirely within the stem of *A. trijuga*, i.e., it is completely endogenous (Figure 4 and Figure S2). Some EPs increase in volume due to a rise in cell number, where the cells dedifferentiate and constitute the floral meristem (Figure 4A,B). These endophytic patches exhibit radial polarity; the cells situated in the inner part (oriented towards the center of the *A. trijuga* stem) retain the characteristics described above, featuring prominent nuclei and dense cytoplasm. In contrast, the cells of the EP oriented towards the cortex and the periderm of the host stem enlarge, with the cytoplasm becoming less dense and developing vacuoles; consequently, the nucleus appears small relative to the overall cell size (Figure 4B). This polarized PD develops in a broad and flattened floral meristem (Figure 4C). Floral organs are initiated on the flanks of the apex; the whorls of the perianth organs are initiated first (Figure 4D).

Host cells contacting floral buds collapse, gradually forming a small chamber where floral meristem and flowers develop (Figure 4C,D and Figure S2A–C). The floral meristem develops into either a staminate or pistillate flower. Before the flower emerges, all the floral organs (gynoecium or androecium) are fully formed (Figure 4 and Figure S2). The developing flower grows through the periderm and emerges on the surface of the stem of the *A. trijuga* plant (Figure 4E–G and Figure S2).

The unisexual flowers are distributed on different branches of the same individual of *A. trijuga*. Although in the section of the *A. trijuga* stem parasitized by *P. berteroi*, floral buds emerge in the upper portion while old flowers or fruits are located in the basal region (Figure 1D–F), examination of the sections reveals that there are flowers at various stages of development intermixed throughout (Figure 4E,F). Flowers emerge perpendicularly to the *A. trijuga* branch (Figure 4D–G).

### 2.5. Sinkers of Flowers

**Figure 5:**
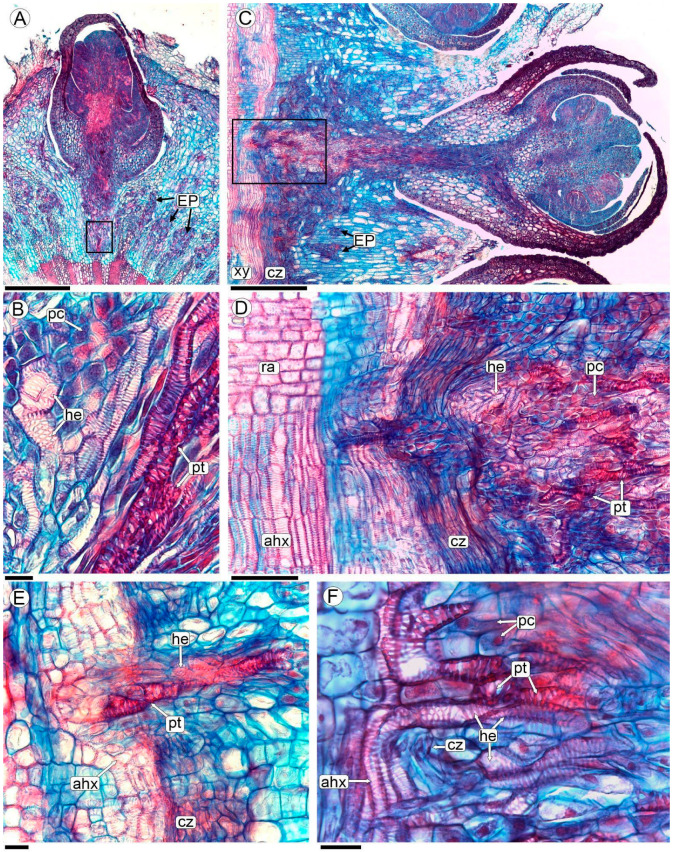
Sinker formation in *P. berteroi* flowers in transverse (**A**,**B**) and longitudinal radial section (**C**–**F**), analyzed with LM. (**A**–**D**) Flowers of *P. berteroi* and their sinkers; (**B**) detail of the sinker showing parasite and host tracheary elements separated by parenchymal cells, corresponding to the box indicated in photo (**A**); (**D**) close-up of the plate in the radial longitudinal section of the stem, corresponding to the boxed area in (**C**); (**E**) detail of the cambial zone showing the sinker with the *A. trijuga* tracheary elements and the tracheoids of *P. berteroi*; (**F**) another detail showing the *A. trijuga* vessels of the axial system and the tracheary elements entering the sinker. The tracheary elements of *A. trijuga* and the tracheoids of *P. berteroi* are in contact. Abbreviations: ahx: axial host xylem; cz: cambial zone; EP: endophytic patches; he: host tracheary elements; pc: parenchyma cell; pt: parasite tracheoid; ra: rays; xy: xylem. Scales: (**A**) 200 µm; (**B**,**E**,**F**) 20 µm; (**C**) 500 µm; (**D**) 100 µm.

The formation of a peg-like extension, the sinker, can be observed at the base of each flower. The sinkers connect the flowers of *P. berteroi* with the cambium-adjoining xylem of the host (Figure 4F, Figure 5A,B, Figures S2 and S3). In differentiated flowers, the sinker has a greater volume, consisting of abundant parasite parenchyma and intermingled xylem elements belonging to both species (Figure 5B–F and Figure S3F).

**Figure 6:**
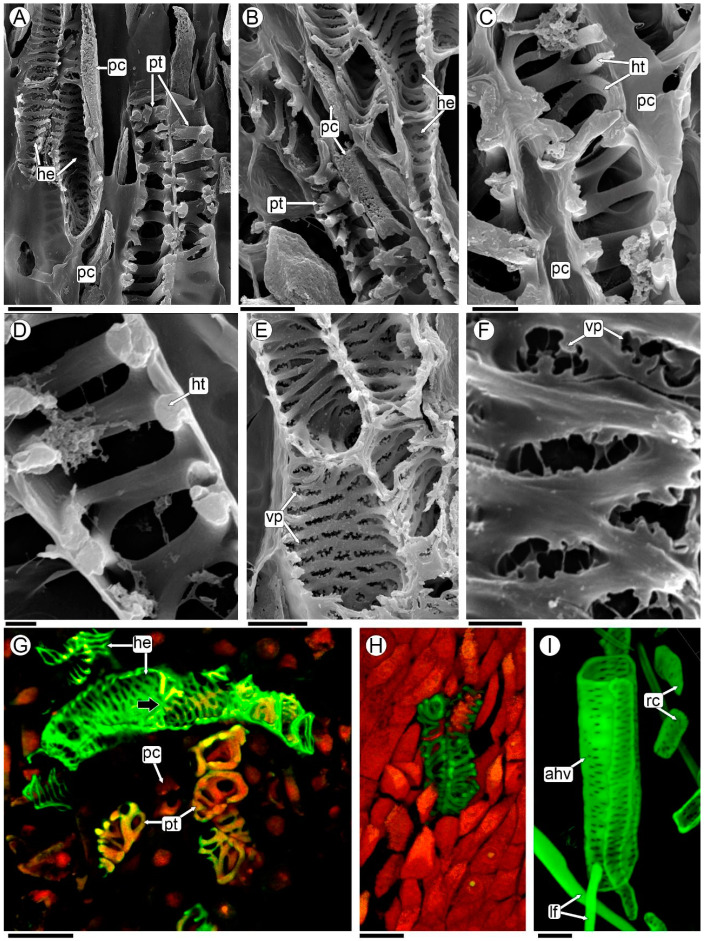
Host vessels and parasite tracheoids of sinkers analyzed with SEM (**A**–**F**), and CLSM maximum images projection of 30 optical sections at 0.95-µm intervals (**G**–**I**). (**A**,**B**) Parasite tracheoids and host tracheary elements separated by parenchymatic cells; (**C**) parasite tracheoids and parenchymatic cells; (**D**) detail of tracheoid with smooth ridges; (**E**,**F**) host tracheary elements with vestured pits; (**G**,**H**) CLSM merged two channels images, green: ex/em 503/508–633; red: 627/635–750; (**G**) detail of sinker highlighting elements of the parasite and host xylem; (**H**) tracheoids from the flower of *P. berteroi*; (**I**) detail of macerated host stem secondary xylem axial elements from a region not invaded by the parasite, CLSM green: 503/508–633 nm. Abbreviations: ahv: axial host vessel; lf: libriform fibers; he: host tracheary elements; ht: helical thickenings; pc: parenchyma cell; pt: parasite tracheoids; rc: rays cells; vp: vestured pits. Scales: (**A**,**B**,**E**) 10 µm; (**C**) 5 µm; (**D**,**F**) 2 µm; (**G**–**I**) 20 µm.

The xylem elements of *P. berteroi* in the sinkers are here described as tracheoids (refer to the discussion section for the use of this term). They are short cells, and the wall is thickened in a helical to scalariform pattern with smooth ridges and lack perforation plates (Figure 5B,E,F, Figure 6A–D,G, and Figure S3D–F). They are almost always oriented with their long axis parallel to the sinker of the flower (Figure 5C,D and Figure S3F).

Conversely, the xylem elements of *A. trijuga* that form the sinker are tracheary elements. They are shorter than the vessels of the axial system of the wood but have the same lateral wall design as the vessels, characterized by scalariform walls with vestured pits (Figure 5B,F and Figure 6A,B,E–G). The *A. trijuga* tracheary elements in the interior of the sinkers are almost always oriented with their longitudinal axis parallel to the sinkers, as are the tracheoids of the *P. berteroi* (Figure 5D,E). This means the *A. trijuga* xylem elements inside the sinkers are perpendicular to those of the secondary xylem of the stem (Figure 5D–F and Figure S3F).

The sinkers do not penetrate the xylem of the *A. trijuga* stem, remaining confined to the cambial zone (Figure 5D,E and Figure S3). Although the sinkers develop entirely within the secondary phloem of the *A. trijuga* stem, a detailed analysis of the cambium and underlying xylem shows that some tracheal elements are reoriented from the secondary xylem of *A. trijuga* and extend into the sinkers (Figure 5D–F). This region exhibits an apparent disruption of the cambium. The vascular elements of *A. trijuga* in the secondary xylem are not invaded by the parasite, exhibit a larger diameter, and are perfectly aligned longitudinally with the stem (Figure 5D).

Using a light microscope, *P. berteroi* tracheoids within the sinkers can be distinguished from *A. trijuga* tracheary elements by their more intensely stained walls (Figure 5B–F and Figure S3D–F). The xylem elements of the two species in the sinkers are typically separated by at least one layer of elongated parenchyma cells (Figure 5B, Figure 6A–C, and Figure S3E,F). However, in some zones, the tracheary elements come into direct contact (Figure 5F and Figure S3D). These parenchymatous cells have a small diameter and an elongated shape, parallel to the vascular elements. However, the presence of a dense cytoplasm allows us to assume that they belong to *A. trijuga*.

The elements of the xylem of *P. berteroi* are always tracheoids (Figure 6G,H), both those in the sinker and those inside the flowers. The vessel walls of *A. trijuga* located inside the sinkers have scalariform intervessel pits, which are arranged in a ladder-like series (Figure 6E–G). Vestures are present in the pits and helical thickenings of the vessel walls. The vestures are tiny protuberances on the secondary wall of the vessel. These ingrowths are only visible using the SEM (Figure 6E,F). The xylem vessels of the axial system of the stem of *A. trijuga* are longer than those of the sinkers and have numerous intervascular pits, alternate or opposite (Figure 6I).

## 3. Discussion

The family Apodanthaceae, together with the Cytinaceae, Mitrastemonaceae, and Rafflesiaceae are classified as endoparasitic plants, so named because their vegetative body is reduced to filaments of parenchyma cells that grow inside their host [6,8,13]. This mode of vegetative body development was named the “endophytic system” by Thoday and Johnson [46], to describe the body of the endoparasite *Arceuthobium pusillum* Peck (Santalaceae). It is only the appearance of the flowers or the fruit scars that makes the presence of the parasite visible [2,47]. Given the peculiarity of this vegetative development, several authors have dealt with the subject in depth [6,15,16].

The endoparasitic *P. berteroi* are notable for invading only those parts already in secondary development. The endophyte’s presence and the emergence of flowers are consistently observed at the same distance from the apex of *A. trijuga* branches. This phenomenon was initially described in dwarf mistletoes (*Arceuthobium*) as “isophasic parasitism”, meaning it grows at the same rate as its host [48]. This pattern aligns with observations in other species of the *Pilostyles* genus [14,25,26,29]. Despite this isophasic growth, in *P. berteroi* the floral primordia and flowers at anthesis are observed together in the apical region of the infested zone. This phenomenon could be explained by the structure of the endophyte, characterized by multiple endophytic patches capable of generating floral meristems, or even by the presence of multiple individuals of *Pilostyles* parasitizing the same branch of *Adesmia*. However, this last hypothesis cannot be corroborated with the methodology used in the present study.

In general terms, the anatomy of healthy wood of *A. trijuga* agrees with that described for other species (*A. boronioides*, [44], *A. rahmeri,* and *A. spinosissima* [45]). None of these studies included electron microscopy observations, nor the structure of the cambium or phloem. The presence of vessels with vestured pits in *A. horrida* Hook. and Arn. that are similar to those described in other Fabaceae, as well as the deformation of the cambium and wood due to environmental variations, has been studied [49]. Modifications in the wood of *Mimosa* species caused by *P. blanchetii* were studied [23], in which it was found that parasitized plants exhibited smaller vessels and characteristics associated with reduced total hydraulic conductivity. The effects produced by *P. blanchetii* on *M. maguirei*, including host architecture and reproductive performance, were analyzed by other authors [50,51]. In *A. trijuga* parasitized by *P. berteroi,* we describe changes in the cambium leading to increased parenchyma formation at the expense of vessel and libriform fibers production. To verify whether the presence of the parasite affects the life and normal development of *A. trijuga* both structurally and physiologically, new collections and in-depth studies are needed.

In the genus *Pilostyles*, the anatomical features of the endophytic system have been examined in several species, including *P. blanchetii* [23,27], *P. hamiltonii* C.A. Gardner [31], *P. haussknechtii* Boiss. [25], and *P. thurberii* A. Gray [29,30]. Although various species of *Pilostyles* parasitize several fabacean genera, the studies referenced above highlight their distinct common characteristics in terms of endophytic structures. First, parasite cells are easily recognized by their large nuclei, usually with two nucleoli, and dense cytoplasm. Second, these cells invade only the parts of the plants that have undergone secondary growth. Finally, the cells that form the vegetative body of *Pilostyles* are organized into an endophytic system consisting of endophytic patches of cells growing exclusively between the elements of the secondary phloem of its host. These EPs are composed exclusively of parenchyma cells without xylem or phloem elements.

The only previous study of *P. berteroi* growing on *A. trijuga* has been published by Kummerow [28], who briefly described the endophyte restricted to the cortex of the host, consisting of cells rich in cytoplasm and with large nuclei. In contrast to Kummerow [28], we found that the endophytic patches of *P. berteroi* are located among the secondary phloem elements of *A. trijuga*. Kummerow [28] states that these descriptions are based on the results of previous studies of *P. blanchetii* and *P. hausknechtii* by Endriss [27] and Solms-Laubach [25,26], respectively. Our observations of the endophytic body of *P. berteroi* are consistent with previous studies on other species of this genus.

The cells of the endophytic system of *P. berteroi* demonstrate characteristics typical of elevated metabolic activity, similar to those observed in meristematic or secretory cells [52,53,54]. These cell types also share several common features that support their specialized functions. They typically exhibit a rapid rate of division and growth, which is essential for their functions. Their dense cytoplasm is rich in organelles that support cell division and facilitate a high level of metabolic activity, enabling efficient nutrient extraction from their host. Additionally, these cells show active transcriptional metabolism, allowing for the rapid production of mRNA and proteins necessary for their specialized roles. Their cell walls may also be thinner to facilitate nutrient absorption and transport. Overall, these features enable them to respond quickly to environmental changes and fulfill their biological functions efficiently. These traits have been observed and discussed for Rafflesiaceae [16] and endoparasitic plants in general [6]. It was even possible to appreciate the presence of double nucleoli in the cells of *P. berteroi*, a characteristic previously mentioned for *P. thurberii* [30].

In all *Pilostyles* species studied, the flower origin was described as endogenous. The mass of tissue embedded in the host phloem that gives rise to flowers was described as the “floral cushion” [25]. Contrary to earlier reports [25,27,31], our observations of *P. berteroi* revealed the polarization of endophytic patches and the subsequent formation of floral meristems from these patches. Kuijt [30], studying *P. thurberii*, was the only author to show some degree of specialization of the endophyte. He recognized three cell types in larger cortical strands and radial sinkers using transmission microscopy. Two cell types are distinguished by the density of cytoplasm and degree of vacuolation, while a sieve element identifies the third cell type. This author did not observe xylem elements in sinkers, nor did he recognize the histology of those portions of the endophyte that give rise to floral meristems. We were unable to identify such cell types in the EPs or sinkers of *P. berteroi* with the techniques used in the present study. However, our observations have shown the same difference in vacuolation that identifies two cell types in the EP that give rise to floral meristems. The study carried out here did not allow recognition of the sieve elements described; additional studies that identify callose using fluorescence, for example, will be necessary.

It is only after the floral meristem is formed and develops whorl primordia that the connection with the xylem of the host is established. These connections were named sinkers [55,56,57]. Kuijt [57], defined them as “a portion of an endophytic system which extends radially into host tissues” and cited them, especially for the genus *Pilostyles* [29]. The formation of sinkers is a common feature of *Pilostyles* parasitism, previously described for other species [23,25,27,28,29,31]. In the *Pilostyles* species examined in the studies cited above, it was possible to describe that the sinkers penetrate the xylem of the host, often following a ray. In the analysis of *P. berteroi* presented here, the sinkers only reach the cambium, without penetrating the xylem of *A. trijuga*. Kummerow [28] states that he has not been able to prove the presence of sinkers (in German: *Senker*), only that he has seen individual cell threads that have broken through the cambium and penetrated the xylem or ray. Although the images in the study do not show structures consistent with sinkers, they provide details of the parasitic cells penetrating a ray of the host’s xylem [28].

The sinkers of *P. berteroi* have a peculiar structure; they are composed of parenchyma and tracheoids of *P. berteroi* mixed with tracheary elements of *Adesmia*. These vascular elements could be in direct contact or separated by parenchyma cells. Several authors have reported the presence of tracheary elements in sinkers of *Pilostyles*, always discussing whether they are tracheids or modified vessels [21,23,25,26,27,29]. In his study of *P. berteroi*, Kummerow [28] mentions the presence of so-called tracheids (in German: *Tracheiden*) only for the “large haustorial complexes”. According to Kummerow, the “large haustorial complexes” refer to the connections between the flowers and the host’s xylem, which he clarifies he did not observe [28]. However, citing other authors, he references the presence of tracheids and illustrates them specifically in the petals [28]. In another study being carried out on *P. berteroi* flowers, the presence of tracheids in the different floral parts has been confirmed [38].

According to the current bibliography, we chose to name the tracheary elements in the sinker of *P. berteroi* as tracheoids, resembling tracheids in their pitted or spirally thickened walls but differing in their form, size and general topography [54,55]. Tracheoids are common elements at vein endings, either in foliar leaf laminae or vascular terminals of nectaries [58,59,60]. They differ from vessels in the absence of perforation plates and from true tracheids in that they are shorter and wider. Tracheoids can occur either as isolated cells or in clusters. When they are found as isolated cells surrounded by parenchyma tissue, they are referred to as tracheoid idioblasts [57]. Alternatively, tracheoids may form clusters within the plant tissue [61].

These cells are also described as being associated with parenchyma in transfusion tissue in several organs, such as staminodes and petals [62], hilum of seeds of faboidean species [63], stylopodium in *Bulbostylis* (Cyperaceae) fruits [64], and they even occur outside the vascular bundles in diverse tissues of cacti [61], epiphytic orchids [65], and ferns [66]. Despite being found in various locations, it has been hypothesized that tracheoids function as water storage cells, or to maximize apoplastic transport, like in Orchidaceae [67]. Considering that the holoparasite requires both nutrients from the host phloem, with which it is in intimate contact, and water, it is hypothesized that tracheoids here play the role of increasing apoplastic transport, which will be addressed in future studies.

This is the first time that tracheoids are described together with host tracheary elements in the haustorial connection between a *Pilostyles* species and its host. The cells of *A. trijuga* within the sinkers are quite sinuous and the perforation plates are difficult to discern; therefore, we decided to name them as “tracheary elements”. Using SEM and CLSM, it was easy to distinguish between the parasite tracheoids and the host tracheary elements. In addition, the presence of vestured pits in the tracheary elements of *Adesmia* is a characteristic feature of fabaceans [68,69]. This confirms that they belong to *Adesmia*.

The vascular cells in the sinkers of the *P. berteroi*/*A. trijuga* system (tracheoids and tracheary elements), and the parenchyma cells between them, form a special type of transfusion tissue. This structure is consistent with the historical and histological definition of a chimera, “which are formed from conglomeration cells that originated from separate zygotes” [70]. Chimeras have been previously described to exist in other holoparasites [4,71]. The interface between host and parasite in the tubers of the Balanophoraceae shows two main patterns, although there are also intermediate forms. In some species, the interface is discrete. The boundaries of the two individuals are well defined, as in *Helosis* and *Lophophytum* [72,73]. In other genera, such as *Balanophora* and *Langsdorffia*, the host/parasite interface is diffuse, and the vascular tissues of the host penetrate the tuber of the parasite, forming chimeric vascular filaments composed of parasite and host cells [74,75,76,77]. In *P. berteroi*, the sinkers are a highly modified chimeric structure, similar to that described in the tuberous organ of Balanophoraceae. However, there is no tissue hypertrophy in the *P. berteroi*/*A. trijuga* system leading to the formation of the typical galls or woodrose of Balanophoraceae.

The flowers of *P. berteroi* obtain their nutrients and water through these chimeric sinkers, as they have greater needs than the vegetative body of the endophyte. Only further ultrastructural analysis will reveal whether there is a direct or indirect connection (via parenchyma) between the vascular elements of both taxa. Such analysis would also allow us to clarify whether the parenchymal cells described here between vessels and tracheoids are transfer cells as reported in other parasites [78,79].

## 4. Materials and Methods

### 4.1. Plant (Parasite and Host) Material

Plants of *A. trijuga* parasitized by *P. berteroi* were selected from two different populations in the province of Jujuy in Argentina. Approximately twenty branches from five parasitized *A. trijuga* plants were collected and fixed in each location. Additionally, two non-parasitized plants from each site were collected and fixed for anatomical comparison.

Material studied, localities, data of the vouchers, and herbaria. *A. trijuga*: Argentina. Jujuy. Tilcara. 23°33′48.7″ S 65°23′59.7″ W 2562 msm. 9 May 2015. Sato HA 444 (JUA). Idem 4 December 2015. Gonzalez AM and Sato H 497 (CTES). Idem. Gonzalez AM and Sato H 498 (CTES). Idem. Tumbaya. Volcán. 23°56′18″ S 65°27′47.5″ W 2021 msm. 4 December 2015. Gonzalez AM and Sato H 496 (CTES). Idem. 20 November 2021. Sato HA 480 (JUA). Idem. 20 November 2022. Sato HA 497 (JUA).

### 4.2. Light Microscopy (LM)

Materials were collected and fixed in FAA (formaldehyde, 70% alcohol, glacial acetic acid, 5:90:5) in the field and preserved in the same fixative at room temperature in the laboratory. To assess the degree of colonization by *P. berteroi*, stems of *A. trijuga* with visible parasite flowers were selected. The parasitized stems were divided into three sections: apical (without *P. berteroi* floral buds), median (with flower buds and anthetical flowers), and basal (with old flowers, scars, or fruits). From each section, five stem samples, each 0.3–0.5 cm in length, were collected and processed. Additionally, five samples of similar sizes were taken from non-parasitized *A. trijuga* stems for comparison.

Both healthy and parasitized *A. trijuga* branches were subjected to histological dehydration followed by a tertiary butanol series [80] and then paraffin infiltration [81]. Serial transversal and longitudinal sections were cut with a rotary microtome (Microm HM 330) to a thickness of 10–12 µm. All sections were stained with Safranin–Astra blue [82] and mounted in synthetic Canada balsam (Biopur). A DM LB2 microscope (LEICA) equipped with a digital camera (Leica ICC50 W) was used for observation and photography. The histochemical tests used were Lugol (starch), Sudan III, and Sudan Black (oils, suberin, wax, cutin) and ferric chloride, (tannins, soluble polyphenols) [81,83,84]. The secondary xylem was described in accordance with IAWA requirements and terminology [85].

### 4.3. Scanning Electron Microscopy (SEM)

Paraffin-embedded sections of parasitized *A. trijuga* stems were also processed for SEM. Serial sections of 30 µm thickness were cut and mounted on glass slides using Haupt’s adhesive [86] and dried for 48 h. Sections were deparaffinized in xylol, dehydrated in an ascending series of acetone, and dried to the critical point in CO_2_. To perform critical point drying, the slides used were pre-cut (4 cm in length) so that they could be placed in the chamber of the critical point drying unit (Denton Desk II, Moorestown, NJ USA). The specimens were gold–palladium coated and observed at the Electron Microscopy Service of the UNNE, Corrientes, using a Jeol LV 5800 SEM (Tokyo, Japan) at 20 kV.

### 4.4. Confocal Laser Scanning Microscope (CLSM)

For the analysis by CLSM, two types of preparations were carried out. Paraffin-embedded material cut at 12 μm thickness (see technical LM) was deparaffinized and mounted in pure glycerol without staining. Additionally, samples of the interface between the host and the base of the parasite flowers were excised under stereoscopic microscopy (avg. 5 mm × 5 mm × 5 mm) and subjected to maceration with a solution of hydrogen peroxide (30%), distilled water (DW), and glacial acetic acid (>99.8%, *V*:*V*:*V* = 1:4:5) in the oven (60 °C) for 48 h [87]. The macerated samples were rinsed three times with DW, stained with 1% Safranin O in DW for 20 min and then washed with DW until the wash solution was clear. After staining, small portions of excised samples were put on a glass slide on a drop of pure glycerol, the cells were separated with a needle under a stereomicroscope, and covered by a cover glass.

Confocal images were acquired with a Stellaris 8 White Light Laser (Leica Microsystems, Wetzlar, Germany) inverted confocal microscope using HC PL APO 10× dry (NA 0.4) and 63× oil immersion (NA 1.4) objectives. Excitation/emission wavelengths were set: blue: 405/413–489, green: 503/508–633, red: 627/635–750 nm. The LAS Navigator software (Leica Microsystems, Germany) was used in conjunction with a 63× objective to view and merge tiles into a single image. Images and three-dimensional (3D) reconstructions were performed using Leica LAS X Stellaris Compass software (Leica Microsystems, Germany). Serial optical sections were taken in the xyz mode to the specimens in three dimensions (3D), from the surface down to the required depths.

## 5. Conclusions

This study provides an in-depth analysis of the characteristics of the endophyte, the origin of the flowers, and the structure of the chimeric sinkers of *P. berteroi* growing inside *A. trijuga* stem. These characteristics have not been studied in such detail before. This is in contrast to the earlier work by Kummerow [28], which only gave a brief overview of these features. There is considerable uniformity of characters among the various species of the genus that have been studied, as noted in a study of the flowers of *P. berteroi* (Romero MF, Sato H and Gonzalez AM, unpublished manuscript]. The different species subject to the most extensive study (*P. boyacensis*, *P. blanchetii*, and *P. berteroi*) show a striking convergence of floral and embryological characteristics. The extensive reduction in holoparasites results in a lack of consistent morphological characters for taxonomic diagnose [21,27,37]. Consequently, the host taxon has been included as a criterion for distinguishing *Pilostyles* species [14]. In this study, several previously undescribed differences were identified, prompting the following question: Does the host determine the structure of the parasite? It appears that the host species indeed influences the structure of the endophyte. The data collected so far suggest new research directions, and a future comparative study of all hosts and the different *Pilostyles* species that parasitize them could help answer the questions raised by these fascinating plants.

## Data Availability

The research data are available at https://ri.conicet.gov.ar/handle/11336/215262, date deposited 18 October 2023.

## Supplementary Materials

The following supporting information can be downloaded at: https://www.mdpi.com/article/10.3390/plants13213010/s1. Figure S1: Stems of *A. trijuga* parasitized by *P. berteroi*. (A) Tangential section of a stem in the initial stage of infestation. Transversal (B–D) and tangential sections (E,F) of older stems showing changes in the cambial zone and mp. Abbreviations: ahv: axial host vessel; cz: cambial zone; EP: endophytic patches; he: host tracheary element; lf: libriform fibers; lra: lignified rays; nra: non-lignified rays; pe: periderm; ph: phloem; xy: secondary xylem. Scales:  A,B,E,F: 100 µm; C,D: 10 µm. Figure S2: Stems of *A. trijuga* showing floral primordia and flowers at various stages of development. Abbreviations: ch: collapsed host cells; co: cortex; cz: cambial zone; EP: endophytic patches; fb: flower bud; pe: periderm; sk: sinker; xy: xylem. Scales: A: 20 µm; B,C: 100 µm; D: 0.5 mm. Figure S3: Host tracheary elements and parasite tracheoids. (A–C) Tracheoids from the flower of *P. berteroi*, and CLSM images of maximum projection of 70 optical sections at 0.45-µm intervals (green: 503/508–633, red: 627/635–750 nm); (A) image digitally colored in red; (B) image digitally colored in green; (C) overlaid images from (A,B). (D–F) LM images of sinkers showing parasite tracheoids and host tracheary elements intermixed, in contact or separated by parenchymatic cells. Abbreviations: ca: cambium; hv: host tracheary elements; pc: parenchyma cell; pt: parasite tracheoids; tr: tracheids. Scales: A–E: 20 µm; F: 50 µm.
