# Supplementary material for: Exploring Hidden Connections: Endophytic System and Flower Meristem Development of Pilostyles berteroi (Apodanthaceae) and Interaction with Its Host Adesmia trijuga (Fabaceae)
Source: Plants (Basel). 2024 Oct 28;13(21):3010. doi: 10.3390/plants13213010 (PMC11548428; doi:10.3390/plants13213010)
Supplement: Supplementary file 1 [file plants-13-03010-s001.zip › plants-3240301-supplementary.pdf]

plants

Article

Exploring Hidden Connections: Endophytic System and Flower Meristem Development of *Pilostyles berteroi* (Apodanthaceae) and Interaction with Its Host *Adesmia trijuga* (Fabaceae)

Ana Maria Gonzalez [1,*,†], María Florencia Romero [1,†] and Héctor A. Sato [2]

[1] Instituto de Botánica del Nordeste (UNNE-CONICET), Facultad de Ciencias Agrarias, Universidad Nacional del Nordeste, Corrientes, PC 3400, Argentina; mariafloromero@gmail.com
[2] Centro de Estudios e Investigaciones Botánicas, Facultad de Ciencias Agrarias, Universidad Nacional de Jujuy, Jujuy, PC 4600, Argentina; hector.a.sato@fca.unju.edu.ar
* Correspondence: anagonzalez.ibone@gmail.com.
† These authors contributed equally to this work.

Supplementary Materials: The following supplemental materials are available at: www.mdpi.com/xxx/s1.

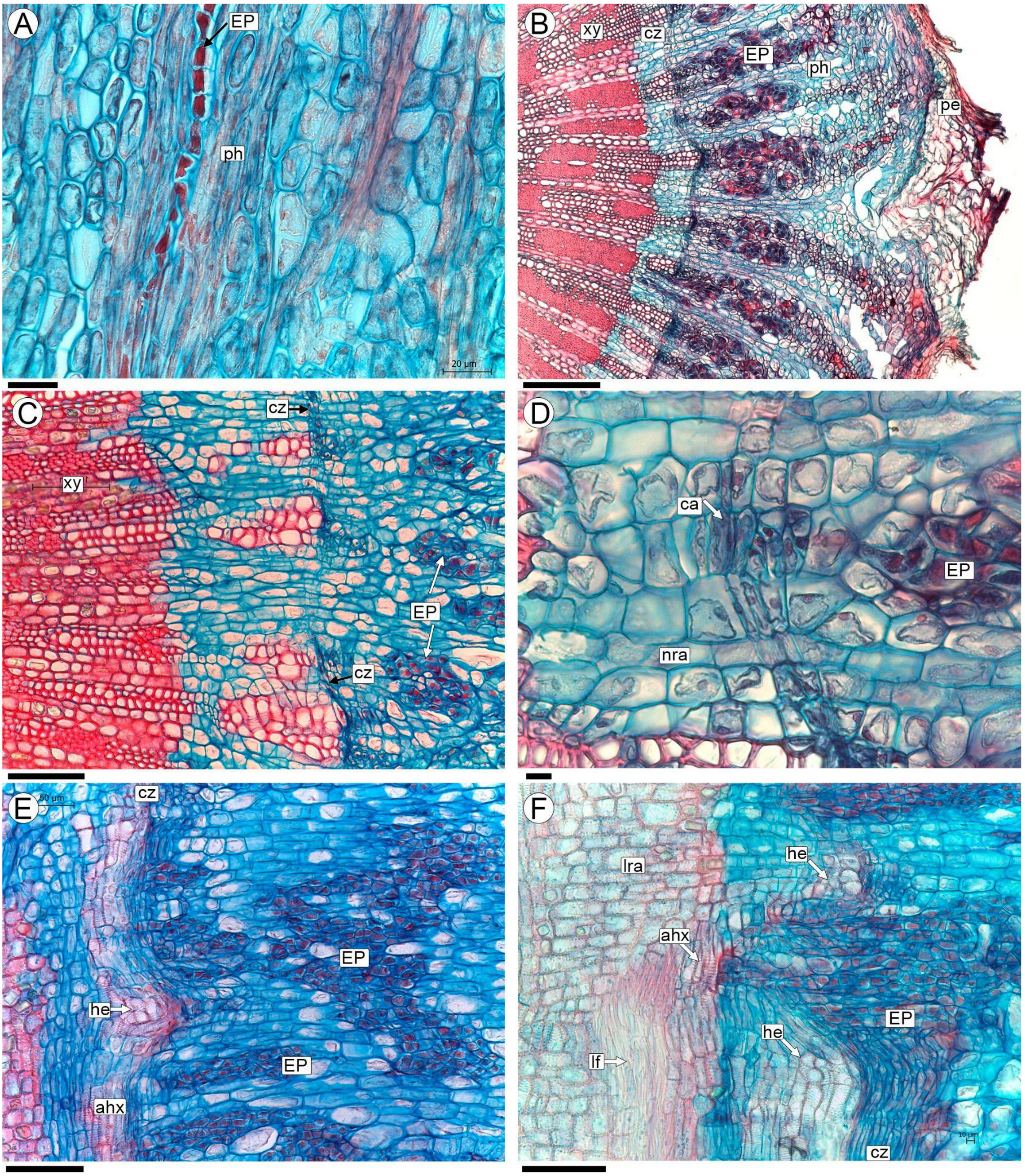

Figure S1: Stems of *A. trijuga* parasitized by *P. berteroi.* (A) A tangential section of a stem in the initial stage of infestation. Transversal (B-D) and tangential sections (E-F) of older stems show changes in the cambial zone and EPs. Abbreviations: ahv: axial host vessel; cz: cambial zone; EP: endophytic patches; he: host tracheary element; lf: libriform fibers; lra: lignified rays; nra: non-lignified rays; pe: periderm; ph: phloem; xy: secondary xylem. Scales: A-B, E-F: 100 µm; C-D: 10 µm.

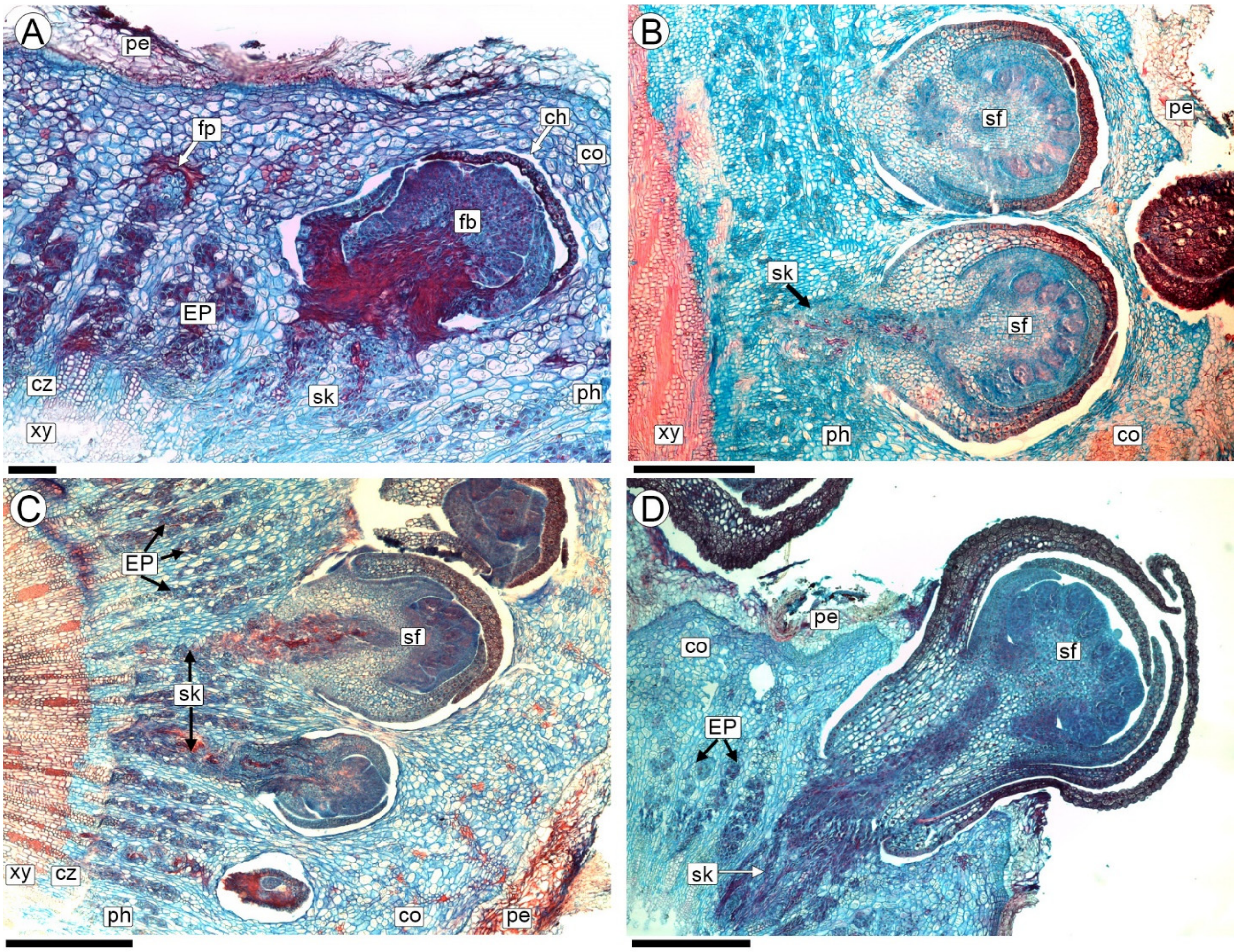

Figure S2: Stems of *A. trijuga* showing floral primordia and flowers at various stages of development. Abbreviations: ch: collapsed host cells; co: cortex; cz: cambial zone; EP: endophytic patches; fb: flower bud; pe: periderm; sk: sinker; xy: xylem. Scales: A: 20 µm; B-C: 100 µm; D-F: 0.5 mm.

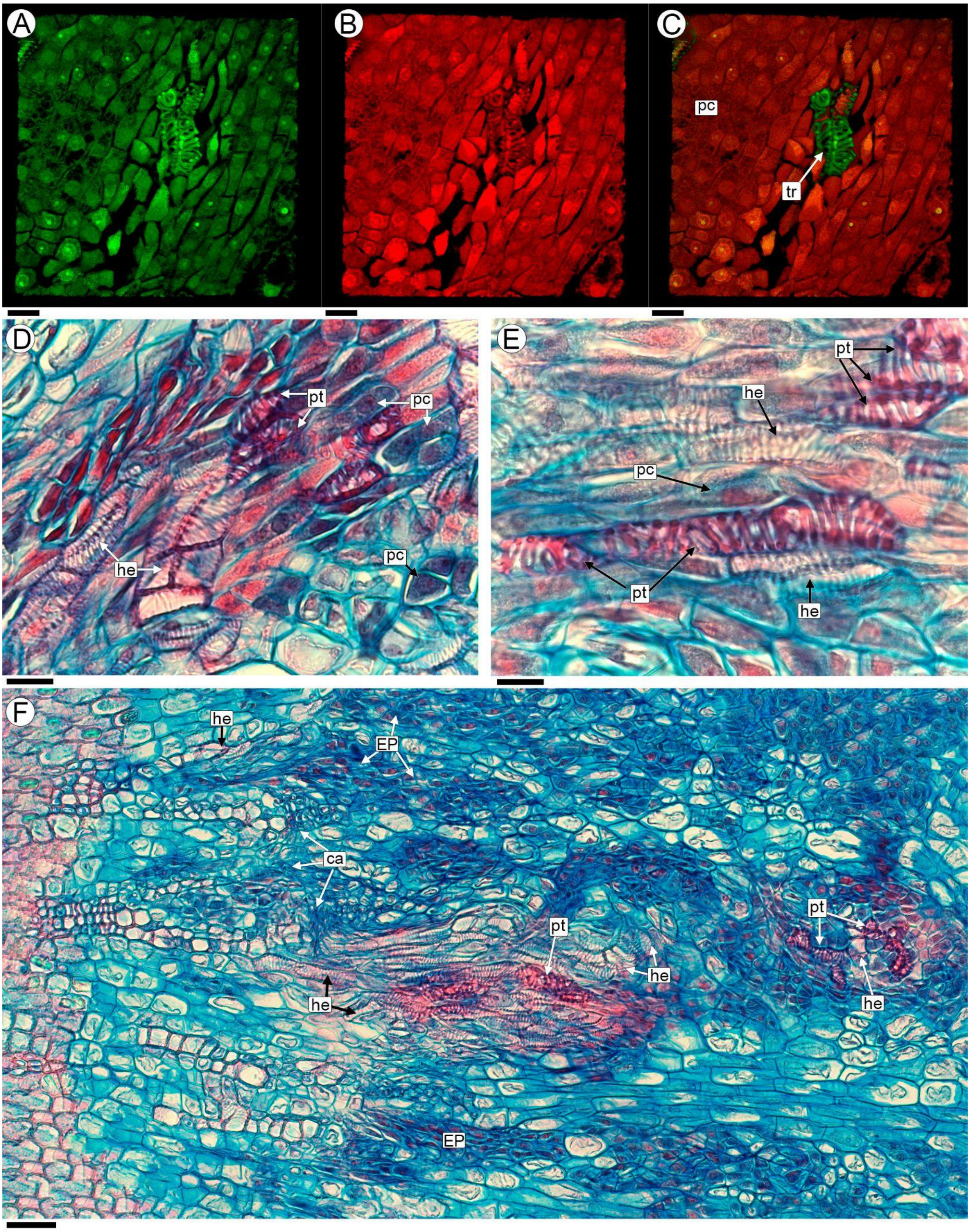

Figure S3: Host tracheary elements and parasite tracheoids. (**A-C**) Tracheoids from the flower of *P. berteroi* and CLSM images of maximum projection of 70 optical sections at 0.45-µm intervals (green: 503/508–633, red: 627/635-750nm);(**A**) image digitally colored in red; (**B**) image digitally colored in green; (**C**) overlaid images from Figures (A) and (B). (**D-F**) LM images of sinkers showing parasite tracheoids and host tracheary elements intermixed, in contact or separated by parenchymatic cells. Abbreviations: ca: cambium; he: host tracheary elements; pc: parenchyma cell; pt: parasite tracheoids; tr: tracheids. Scales: A-E: 20 µm; F: 50 µm
